# Management of multifactorial idiopathic epilepsy in EL mice with caloric restriction and the ketogenic diet: role of glucose and ketone bodies

**DOI:** 10.1186/1743-7075-1-11

**Published:** 2004-10-19

**Authors:** John G Mantis, Nicole A Centeno, Mariana T Todorova, Richard McGowan, Thomas N Seyfried

**Affiliations:** 1 Biology Department, Boston College, Chestnut Hill, MA, USA

## Abstract

**Background:**

The high fat, low carbohydrate ketogenic diet (KD) was developed as an alternative to fasting for seizure management. While the mechanisms by which fasting and the KD inhibit seizures remain speculative, alterations in brain energy metabolism are likely involved. We previously showed that caloric restriction (CR) inhibits seizure susceptibility by reducing blood glucose in the epileptic EL mouse, a natural model for human multifactorial idiopathic epilepsy. In this study, we compared the antiepileptic and anticonvulsant efficacy of the KD with that of CR in adult EL mice with active epilepsy. EL mice that experienced at least 15 recurrent complex partial seizures were fed either a standard diet unrestricted (SD-UR) or restricted (SD-R), and either a KD unrestricted (KD-UR) or restricted (KD-R). All mice were fasted for 14 hrs prior to diet initiation. A new experimental design was used where each mouse in the diet-restricted groups served as its own control to achieve a 20–23% body weight reduction. Seizure susceptibility, body weights, and the levels of plasma glucose and β-hydroxybutyrate were measured once/week over a nine-week treatment period.

**Results:**

Body weights and blood glucose levels remained high over the testing period in the SD-UR and the KD-UR groups, but were significantly (p < 0.001) reduced in the SD-R and KD-R groups. Plasma β-hydroxybutyrate levels were significantly (p < 0.001) increased in the SD-R and KD-R groups compared to their respective UR groups. Seizure susceptibility remained high in both UR-fed groups throughout the study, but was significantly reduced after three weeks in both R-fed groups.

**Conclusions:**

The results indicate that seizure susceptibility in EL mice is dependent on plasma glucose levels and that seizure control is more associated with the amount than with the origin of dietary calories. Also, CR underlies the antiepileptic and anticonvulsant action of the KD in EL mice. A transition from glucose to ketone bodies for energy is predicted to manage EL epileptic seizures through multiple integrated changes of inhibitory and excitatory neural systems.

## Background

Epilepsy is a neurological disorder involving recurrent abnormal discharges of neurons that produce epileptic seizures [1]. With the exception of stroke, epilepsy is one of the most prevalent human neurological afflictions affecting about 1% of the US population [2,3]. Many persons with epilepsy manifest partial or generalized seizures without symptoms of brain abnormality, i.e., idiopathic epilepsy [1,4,5]. In contrast to idiopathic epilepsy, symptomatic or acquired epilepsy often accompanies brain trauma, injury, or neurostructural defects. While some idiopathic epilepsies are inherited as simple Mendelian traits, most are multifactorial where more than one gene together with environmental factors contribute to the disease phenotype [6,7].

Epilepsy animal models are used widely to test the influence of environmental and genetic factors on seizure mechanisms. The epileptic EL mouse is a natural model for human multifactorial idiopathic epilepsy and was first discovered in 1954 in an outbred DDY mouse colony [6,8-10]. EL mice experience complex partial seizures with secondary generalization similar to those seen in humans [6,10]. Seizures in EL mice commence with the onset of puberty (50–60 days), originate in or near the parietal lobe, and then spread to the hippocampus and to other brain regions [6,11-13]. The seizures are accompanied by electroencephalographic abnormalities, vocalization, incontinence, loss of postural equilibrium, excessive salivation, and head, limb, and chewing automatisms [10,12,14-17]. A reactive gliosis accompanies seizure progression in adult EL mice involving both astrocytes and microglia [18,19]. Epileptic seizures in EL mice also model Gowers' dictum, where each seizure increases the likelihood of recurrent seizures [6]. Seizure susceptibility can be managed with phenytoin and phenobarbital as well as with diet therapies to include the ketogenic diet and caloric restriction [20-22]. Gene-environmental interactions play a significant role in the determination of seizure frequency and onset in EL mice as with multifactorial human idiopathic epilepsies [6,9,23].

Despite intensive antiepileptic drug (AED) research and development, seizures remain unmanageable or refractory in many persons with epilepsy [24-26]. As an alternative to AEDs diet therapies can be effective in the management or control of epilepsy. Fasting has long been recognized as an effective antiepileptic therapy for a broad range of seizure disorders [27-29]. Since fasting produces ketonemia, it was originally thought that ketone bodies (β-hydroxybutyrate and acetoacetate) might underlie the antiepileptic effects of fasting [27,30]. Consequently, high fat, low protein, low carbohydrate KDs were developed to mimic the physiological effects of fasting [25,27,31,32]. Although the KD significantly elevates circulating ketone body levels, later studies showed that ketone bodies alone were unable to account for the antiepileptic and anticonvulsant effects of the KD in humans or in animal epilepsy models [20,31,33-38]. Since the KD manages epilepsy best when administered in restricted amounts and since fasting lowers blood glucose levels, Seyfried and co-workers suggested that caloric restriction might contribute to the antiepileptic and anticonvulsant effects of the KD [21,29,38].

CR is a natural dietary therapy that improves health, extends longevity, and reduces the effects of neuroinflammatory diseases in rodents and humans [21,29,39,40]. CR is produced from a total dietary restriction and differs from acute fasting or starvation in that CR reduces total caloric energy intake without causing anorexia or deficiencies of any specific nutrients [38]. In other words, CR extends the health benefits of fasting while avoiding starvation. Besides improving health, CR has both antiepileptic and anticonvulsant effects in EL mice and in other animal epilepsy models [20,21,41]. A reduction in blood glucose with a corresponding elevation in blood ketone bodies is thought to underlie the antiepileptic and anticonvulsant effects of CR [21,29,38].

Glucose uptake and metabolism increases more during epileptic seizures than during most other brain activities [42-44]. Blood glucose also positively correlates with flurothyl-induced seizures in rats and high glucose may exacerbate human seizure disorders [45]. Neuronal excitability and epileptic seizures are directly related to rapid glucose utilization and glycolysis [42,43,45-51]. It is not yet clear, however, to what extent enhanced glycolysis is related to the cause or effects of seizure activity [29]. Nevertheless, a transition in brain energy metabolism from glucose utilization to ketone body utilization reduces neural excitation and increases neural inhibition through multiple integrated systems [29,38]. Based on these and other observations [50,52-54], we proposed that many epilepsies, regardless of etiology, might ultimately involve altered brain energy homeostasis [29].

In this study, we compared the antiepileptic and anticonvulsant effects of both the KD and CR in adult EL mice that experienced at least 15 recurrent complex partial seizures. The results show that seizure control in EL mice is more associated with the amount than with the origin of dietary calories, and that CR underlies the antiepileptic and anticonvulsant action of the KD in EL mice. A preliminary report of these findings was recently presented [55].

## Results

### Diet composition and tolerance

The composition of each diet is shown in Table 1 and in the Methods. No adverse effects of the diets were observed in either R-fed mouse group. Despite the 20–23% body weight reduction, mice in both R-fed groups appeared healthy and were more active than the mice in the UR-fed groups as assessed by ambulatory and grooming behavior. With the exception of oily fur, the KD-fed mice appeared active and healthy throughout the study as previously found [20]. No signs of vitamin or mineral deficiency were observed in the R-fed mice according to standard criteria for mice [56]. These findings are consistent with the well-recognized health benefits of mild to moderate caloric restriction in rodents [57], and support our previous findings that both the KD and a moderate CR are well tolerated by EL mice [20,21].

**Table 1:** Composition (%) of the Standard Diet and the Ketogenic Diet ^1^

**Components**	**Standard Diet (SD)**	**Ketogenic Diet (KD)**
Carbohydrate	62	0
Fat	6	75
Protein	27	14
Fiber	5	12
Energy (Kcal/gr)	4.4	7.8

^1 ^According to manufacturer's specifications (see Methods).

### Influence of caloric restriction on body weight

**Figure 1:**
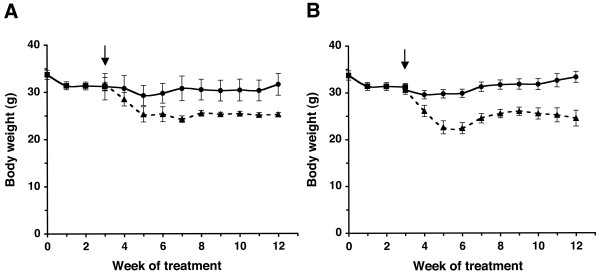
**Influence of diet on body weight in adult EL mice fed the SD (A) or the KD (B). **Squares represent the pre-trial period when all mice were fed the SD-UR. Circles and triangles represent the UR-fed and R-fed groups, respectively. Values are expressed as the mean ± SEM (n = 6 mice per group). Arrow indicates initiation of CR.

All mice were matched for age (approximately 210 days) and body weight (approximately 31.0 ± 1.5 g) before the start of the dietary treatment (Fig. 1). All mice lost approximately 7–9% of their body weight during the 14 hr fast. Body weight remained relatively stable over the nine-week treatment period in both UR-fed mouse groups (Fig. 1). The 20–23% body weight reduction was achieved in the R-fed groups after about two weeks of gradual food restriction. However, more difficulty was encountered initially in maintaining a stable body weight reduction for the KD-R group than for the SD-R group. This difficulty may result from the high caloric content of the KD that produces greater body weight changes per calorie adjustment than the SD. We also estimated that the degree of CR necessary to maintain the 20–23% body weight reduction was about 38–45% for the SD and about 45–52% for the KD.

### Influence of diets on seizure susceptibility in adult EL mice

**Figure 2:**
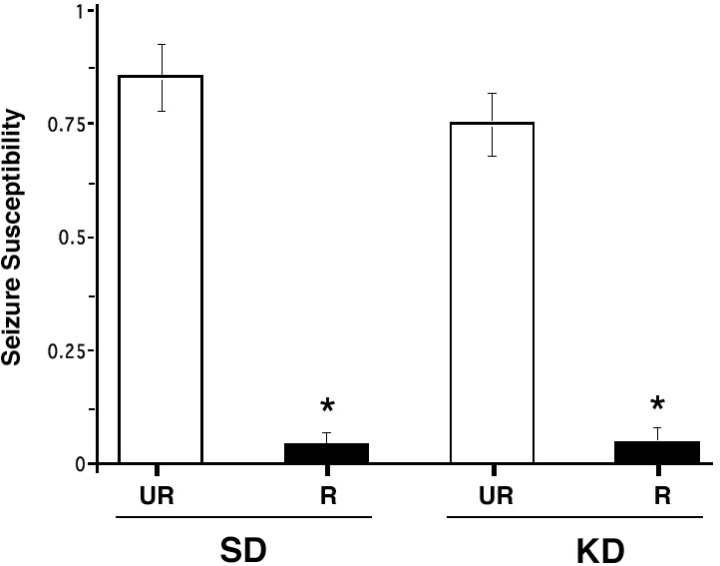
**Influence of diet on seizure susceptibility in adult EL mice. **Asterisks indicate that seizure susceptibility was significantly lower (p < 0.001) in the R-fed groups than in their respective UR-fed groups. Values were pooled from treatment weeks 5–12 (see Fig. 1) and are expressed as the mean ± SEM (n = 6 mice per group).

All mice had at least 15 recurrent seizures before the start of dietary treatment (arrow, Fig. 1). The seizures occurred occasionally during routine cage changing prior to the pre-trial period and regularly from handling during the pre-trial test period. Seizure susceptibility was analyzed in all mouse groups after the R-fed mice achieved a stable body weight reduction, i.e., week five of treatment (Figs. 1 and 2). Seizure susceptibility was high for both UR-fed groups throughout the study. In both R-fed groups, seizure susceptibility decreased from 1.0 to about 0.3 after two weeks and remained significantly lower than that of the UR-fed control groups from treatment weeks 5–12 (Fig. 2). Only a single mouse in the KD-R group had a break-through seizure on week 8. Taken together, our findings show that seizure management in EL mice is more associated with the amount than with the origin of dietary calories.

### Influence of diets on plasma glucose and β-hydroxybutyrate levels

**Figure 3:**
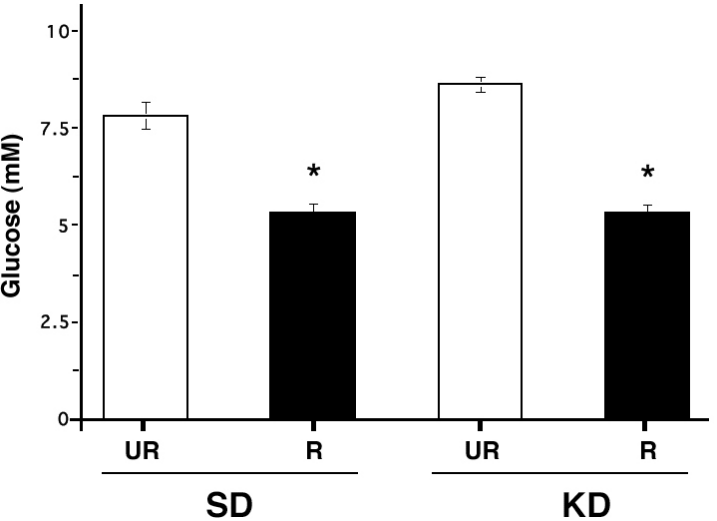
**Influence of diet on plasma glucose levels in adult EL mice. **Asterisks indicate that the plasma glucose levels were significantly lower (p < 0.001) in the R-fed groups than in their respective UR-fed groups. Other conditions are as in Figures 1 and 2.

Plasma glucose levels were analyzed in all mouse groups after the R-fed mice achieved a stable body weight reduction (Figs. 1 and 3). Glucose levels remained high for both UR-fed groups throughout the study and were stable over treatment weeks 5–12. However, plasma glucose levels were somewhat lower (about 8 mM) in both UR-fed groups between treatment weeks 3–5 compared to the pre-trial glucose levels (about 10 mM). This reduction might result from a combination of repetitive handling, seizures, blood collection, and the initial fast. In both R-fed mouse groups, the plasma glucose levels decreased from about 10 mM to about 5.0 mM after three weeks and remained significantly lower than those of their respective UR-fed control groups (Fig. 3).

**Figure 4:**
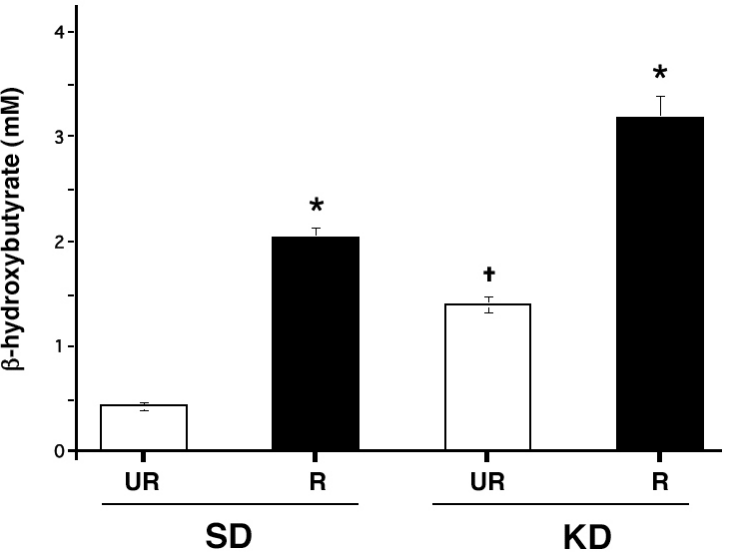
**Influence of diet on plasma β-hydroxybutyrate levels in adult EL mice. **Asterisks indicate that the plasma β-hydroxybutyrate levels were significantly higher (p < 0.001) in the R-fed groups than in their respective UR-fed groups. The cross indicates that the plasma β-hydroxybutyrate levels were significantly higher (p < 0.001) in the KD-UR group than in the SD-UR group. Other conditions are as in Figures 1 and 2.

Plasma β-hydroxybutyrate levels were also analyzed in all mouse groups after the R-fed mice achieved a stable body weight reduction (Figs. 1 and 4). These levels remained low in the SD-UR group throughout the study and were stable for treatment weeks 5–12. β-hydroxybutyrate levels were significantly higher in the R-fed groups than in their respective UR-fed control groups (Fig. 4). These levels were also significantly higher in the KD-UR group than in the SD-UR group. The levels increased from about 0.4 mM to about 1.7 mM in the SD-R group and to about 3.0 mM in the KD-R group. These findings demonstrate that circulating β-hydroxybutyrate levels were inversely related to circulating glucose levels and that elevated β-hydroxybutyrate levels alone are not associated with seizure susceptibility.

### Statistical relationships among variables

**Figure 5:**
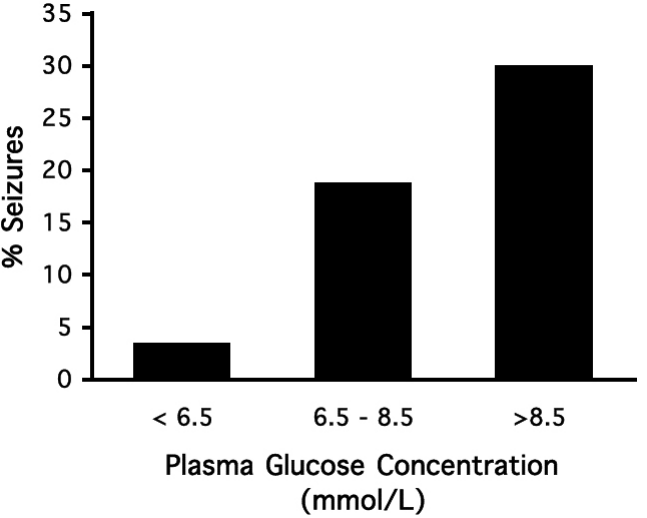
**Association of plasma glucose and seizure susceptibility in adult EL mice. **Data were obtained from all four dietary groups over treatment weeks 3–12 for a total of 234 seizure and glucose measurements. Seizure frequency in the three plasma glucose groups (< 6.5 mmol, 6.5–8.5 mmol, and > 8.5 mmol/L) was 8/234, 44/234, and 70/234, respectively. The association between glucose and seizure susceptibility was highly significant as determined by Chi-square analysis (p < 0.001).

The relationship between body weight, food intake, plasma glucose levels, plasma β-hydroxybutyrate levels, and seizure susceptibility was determined using Pearson bivariate correlation analysis (Table 2). All variables were significantly (p < 0.01) correlated with each other. Positive correlations were found among body weight, food intake, glucose, and seizure susceptibility. On the other hand, β-hydroxybutyrate was negatively correlated with all variables. The correlations among glucose, β-hydroxybutyrate, and seizure susceptibility were also apparent from the data in Figures 2,3,4. Plasma glucose was significantly (p < 0.001) associated with seizure susceptibility in the EL mouse, as determined by chi-square analysis (Fig. 5). These results support our previous findings that glucose levels are predictive of seizure susceptibility in adult EL mice [21,29].

**Table 2:** Pearson bivariate correlation of body weight, food intake, plasma glucose levels, plasma b-hydroxybutyrate levels, and seizure susceptibility in adult EL mice ^1^

**Parameter**	**Body weight (g)**	**Food Intake (Kcal)**	**Glucose (mM)**	**Ketones (mM)**	**Seizure Susceptibility**
Body weight (g)	1.000				
Food Intake (Kcal)	0.488*	1.000			
Glucose (mM)	0.509*	0.382*	1.000		
Ketones (mM)	-0.379*	-0.379*	-0.429*	1.000	
Seizure Susceptibility	0.512*	0.464*	0.616*	-0.510*	1.000

^1 ^Data were obtained from all four dietary groups over the treatment weeks 3–12 for a total number of 210 seizure and glucose measurements (see figure 1).

* All correlations were significant at the 0.01 level (2-tailed).

Binary logistic regression was also used to determine the relationship between seizure susceptibility, plasma glucose, and plasma β-hydroxybutyrate levels when mice were fed either the SD and/or the KD. The data indicate that regardless of diet, glucose could predict seizure susceptibility with an approximate 75 to 78 % accuracy (Table 3). Although β-hydroxybutyrate could also predict seizure susceptibility, we previously showed that β-hydroxybutyrate levels were dependent on and were inversely related to plasma glucose levels [21].

**Table 3:** Binary logistic regression analysis of the maximum likelihood estimates between plasma glucose, and seizure susceptibility in adult EL mice fed either the SD or KD^1^

**Dietary groups**	**Parameter**	**Df**^2^	**B**^3^	**SEM**^4^	**Wald x**^2 5^	**p value**^6^
SD	Glucose	1	0.774	0.139	30.962	0.01
	Constant	1	-0.584	1.013	29.292	0.01
KD	Glucose	1	0.787	0.157	25.033	0.01
	Constant	1	-5.801	1.180	24.177	0.01
Both Diets	Glucose	1	0.752	0.102	54.682	0.01
	Constant	1	-5.507	0.759	52.625	0.01

^1 ^Data were obtained from all four dietary groups over the treatment weeks 3–12 for a total number of 210 individual measurements of plasma glucose and seizure susceptibility.

^2 ^Df, degrees of freedom.

^3 ^B, Estimate of the association between glucose and seizure susceptibility.

^4 ^The estimated error of the mathematical weighting, indicating the precision of the estimated coefficient.

^5 ^The Wald test statistic was computed from the data compared by using x^2 ^distribution with 1 degree of freedom. The test statistic is used to determine the p value.

^6 ^The probability of Type I error.

## Discussion

We found that restriction of either a high carbohydrate low fat standard diet or a high fat low carbohydrate KD was equally effective in reducing seizure susceptibility in adult EL mice with active epilepsy. Moreover, seizure susceptibility remained similarly high in these mice when either diet was fed *ad libitum *or unrestricted. These findings indicate that the KD, when fed unrestricted, is unable to reduce seizure susceptibility in adult EL mice. Although the KD delays epileptogenesis in young seizure naïve EL mice when fed *ad libitum*, the effect is transient [20]. These findings are interesting since previous observations with children suggest that the antiepileptic and anticonvulsant effects of the KD are best when the diet is administered in restricted amounts [25,31]. Indeed, seizure protection is often less in children that gain weight than in those who maintain or reduce body weight on the KD (Freeman, personal communication). Previous studies also indicate that restriction of high carbohydrate diets elevate seizure threshold [58]. Our findings in EL mice support these observations and suggest that CR may be necessary for the antiepileptic and anticonvulsant effects of the KD.

We previously showed that mild to moderate CR delayed epileptogenesis and reduced seizure susceptibility in seizure naïve juvenile and adult EL mice by reducing blood glucose and elevating ketone bodies [21]. Although our data show that circulating β-hydroxybutyrate levels are inversely related to circulating glucose levels, elevated ketone body levels are not directly associated with reduced seizure susceptibility in EL mice. This conclusion derives from the finding that seizure susceptibility is high in the KD-UR mice despite elevated β-hydroxybutyrate levels and from finding that seizure protection was similar in the SD-R and KD-R groups despite significantly higher β-hydroxybutyrate levels in the KD-R than in the SD-R group. These results are consistent with previous studies in EL mice and in non-genetic seizure models that elevated ketone bodies alone are unable to account for the antiepileptic or anticonvulsant action of the KD [20,31,33-38].

Under normal physiological conditions brain cells derive most of their energy from glucose or glucose-derived metabolites, e.g., lactate [46,59,60]. Also, brain glucose uptake is greater during epileptic seizures than during most other brain activities [43]. During fasting or caloric restriction, however, circulating glucose levels fall causing brain cells to rely more heavily for energy on ketone bodies that gradually increase with food restriction [29,61]. It is the transition from glucose to ketone bodies for brain energy that is thought to underlie the antiepileptic and anticonvulsant effects of caloric restriction [29]. Although the KD we used contained no carbohydrates, the mice eating this diet maintained high glucose levels and seizure susceptibility. The persistence of high glucose levels in the KD-UR group would prevent the transition to ketones for energy despite high levels of circulating ketone bodies. Our results show that circulating glucose levels accurately predict seizure susceptibility in EL mice regardless of diet composition or circulating ketone body levels.

We used a new experimental design for caloric restriction in this study. Instead of restricting calories in the R-fed mice based on the average food consumption of the UR control mice as we did previously [21], each R-fed mouse served as its own control to achieve and maintain a 20–23% body weight reduction. We found in a pilot study that isocaloric restriction of the KD was unable to reduce body weight to the same degree as that observed for a similar restriction of the SD. The new experimental design reduces variability in body weights and in caloric intake among mice fed diets widely different in nutritional composition and caloric content. In using body weight, rather than caloric intake, as an independent variable we were able to more accurately measure the statistical associations among circulating energy metabolites and seizure susceptibility. Thus, this type of experimental design is recommended for those studies attempting to evaluate the relationships among nutrition, metabolism, and disease phenotype.

**Figure 6:**
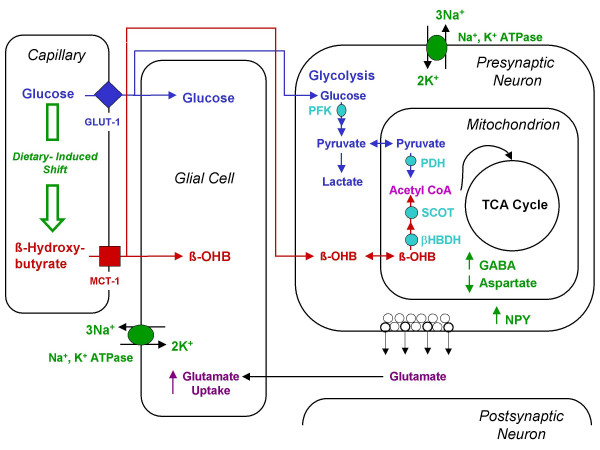
**Perspectives on the metabolic management of epilepsy through a dietary reduction of glucose and elevation of ketone bodies. **A dietary reduction in blood glucose levels will increase ketone utilization for energy. This is expected to shift the neural environment from excitation to inhibition through multiple integrated systems. Abbreviations: GLUT-1 (glucose transporter), MCT (monocarboxylate transporter), PFK (phosphofructokinase), PDH (pyruvate dehydrogenase), SCOT (succinyl-CoA-acetoacetate-CoA transferase), β-OHB (β-hydroxybutyrate), β-HBDH (β-hydroxybutyrate dehydrogenase), NPY (Neuropeptide Y), GABA (gamma-aminobutyric acid). Modified from Seyfried et al., 2004 [38].

We previously discussed the potential mechanisms by which CR might reduce seizure susceptibility [21,29,38]. Some of the cellular systems potentially modulated through CR that could influence brain excitability are illustrated in Fig. 6. We suggest that the transition from glucose to ketone bodies as a major energy fuel for the brain produces multiple changes in gene-linked metabolic networks. It is these changes that gradually adjust neurotransmitter pools and membrane excitability to restore the physiological balance of excitation and inhibition [29]. CR could also influence seizure susceptibility through the neuroendocrine system involving leptin signaling and increased levels of neuropeptide-Y, a peptide with antiepileptic and anticonvulsant effects [62-65]. While the levels of γ-aminobutyric acid (GABA) are increased in synaptosomes via the increased action of glutamic acid decarboxylase during the metabolism of ketone bodies for energy, the levels of aspartate decrease due to the formation of glutamate [66]. In addition, ketone body metabolism could increase membrane ionic pump activity [67,68]. Increased pump activity could increase membrane potential in neurons while also increasing neurotransmitter uptake in glia [29]. We do not exclude the possibility that CR may reduce seizure susceptibility in EL mice through additional mechanisms [31,69]. It is our contention that CR reduces seizure susceptibility through multiple integrated systems providing a multifactorial therapy to a multifactorial disease. Further studies in the EL mouse and in other epilepsy models are needed to identify the exact mechanisms of CR action in managing epileptic events.

## Conclusions

We conclude that seizure susceptibility in EL mice is dependent on plasma glucose levels and that seizure control depends more on the amount than on the origin of dietary calories. Also, we found that CR underlies the antiepileptic action of the KD in EL mice. A transition from glucose to ketone bodies for energy is predicted to manage EL epileptic seizures through multiple integrated changes of inhibitory and excitatory neural systems.

## Methods

### Mice

The inbred EL/Suz (EL) mice were originally obtained from J. Suzuki (Tokyo Institute of Psychiatry). The mice were maintained in the Boston College Animal Care Facility as an inbred strain by brother × sister mating. The mice were group housed (prior to initiation of study) in plastic cages with Sani-chip bedding (P.J. Murphy Forest Products Corp., Montville, N.J.) and kept on a 12-hr light/dark cycle at approximately 22°C. Cotton nesting pads were provided for warmth when animals were individually housed. All cages and water bottles were changed once per week. Only females were used for these studies as adult males die sporadically with age from acute uremia poisoning due to urinary retention [70]. The procedures for animal use were in strict accordance with the NIH Guide for the Care and Use of Laboratory Animals and were approved by the Institutional Animal Care Committee.

### Seizure Susceptibility and Seizure Testing

Seizure onset in EL mice is generally between 60–70 days of age as previously described [6]. These seizures occur occasionally during routine cage changing. Our recently developed seizure handling protocol was used to regularly induce seizure susceptibility in EL mice [6,21]. Briefly, the testing procedure included repetitive handling and simulated the stress normally associated with weekly cage changing, i.e., picking the mouse up by the tail for short intervals and transferring it to a clean cage with fresh bedding. The test included two trials that were separated by 30 min. In each trial, a single mouse was held by the tail for 30 sec at approximately 10–15 cm above the bedding of its home cage. After 30 sec, the mouse was placed into a clean cage with fresh bedding for 2 min. The mouse was then held again for 15 sec before being returned to its home cage. Trial 2 was performed even if the mouse experienced a seizure in trial 1. The epileptic seizures commenced during holding or soon after the mice were placed on the clean bedding. Mice that developed an epileptic seizure while handling were placed immediately in either the clean cage or their home cage depending on the testing stage. Mice were tested each week for a total of 13 measurements over a 12-week period using this method. Mice were undisturbed between testing phases (no cage changing) and testing was performed between 12 to 3 pm.

### Seizure Phenotype

Mice were designated seizure susceptible if they experienced a generalized seizure during seizure testing. Generalized seizures in EL mice involve loss of postural equilibrium and consciousness, together with excessive salivation, head, limb, and chewing/swallowing automatisms. An erect forward-arching Straub tail, indicative of spinal cord activation, was also seen in most mice having generalized seizures. Mice that displayed only vocalization and twitching without progression to generalized seizure were not considered seizure susceptible [6,21]. Seizure susceptibility scores were generated for each mouse according to the seizure severity scores previously described [6]. Mice having a score of 4 or 5 were assigned a susceptibility score of 1.0, whereas mice having a seizure severity score less than 4 were given a susceptibility score of 0. The seizure susceptibility for each mouse was then averaged over multiple tests and the mean seizure susceptibility for a mouse dietary group was determined.

### Diets

All mice received PROLAB RMH3000 chow diet (LabDiet, Richmond, IN, USA) prior to the experiment. This is the standard food pellet diet (SD) and contained a balance of mouse nutritional ingredients. According to the manufacturer's specification, this diet delivers 4.4 Kcal/g gross energy, where fat, carbohydrate, protein, and fiber comprised 55 g, 520 g, 225 g, and 45 g/Kg of the diet, respectively. The ketogenic diet (KD) was obtained from the Zeigler Bros., Inc. (Gardners, PA, USA) in butter-like form and also contained a balance of mouse nutritional ingredients. According to the manufacturer's specification, the KD delivers 7.8 Kcal/g gross energy, where fat, carbohydrate, protein, and fiber comprised 700 g, 0 g, 128 g, and 109 g/Kg of the diet, respectively. The fat in this diet was derived from lard and the diet had a ketogenic ratio (fats: proteins + carbohydrates) of 5.48:1. The individual % composition of each dietary energy component for the SD and KD diet is shown on Table 1.

### Pre-Trial Period

Seizure susceptibility, body weight, and food intake was measured four times over a three-week period in 24 singly caged female EL mice (approximately 210 days of age). All mice received the SD during the pre-trial period and food intake was determined by subtracting the weight of food pellets remaining in the food hopper after one week from the initial amount given (200 g). The difference was then divided by seven to estimate the average daily food intake. Thus, all mice were highly seizure susceptible at the initiation of the diet therapy.

### Dietary Treatment

After the three-week pre-trial period, the mice were placed into four groups (n = 6 mice/group) where the average body weight of each group was similar (about 31.0 ± 1.5 g) (Fig. 1). All mice were then fasted for 14 hr to establish a similar metabolic set point at the start of the experiment (arrow, Fig. 1). The mice in each group were then given one of four diets to include: 1) the standard diet fed *ad libitum *or unrestricted (SD-UR), 2) the KD fed *ad libitum *or unrestricted (KD-UR), 3) the SD restricted to achieve a 20–23% body weight reduction from the pre-trial weight (SD-R), and 4) the KD restricted to achieve a 20–23% body weight reduction from the pre-trial weight (KD-R). Each mouse in the two R groups served as its own control for body weight reduction. Based on food intake and body weight during the pre-trial period, food in the R-fed mouse groups was reduced until each mouse achieved the target weight reduction of a 20–23%. In other words, the daily amount of food given to each R mouse was reduced gradually until it reached 77–80% of its initial (pre-trial) body weight.

The mice in the SD-UR group received 200 g of food in the hopper/week as in the pre-trial period. For mice in the SD-R group, weighed food pellets were dropped directly into each cage for easy access. The KD was administered to the mice in a modified plastic Falcon tissue culture dish (60 mm × 15 mm). The dish edges were shaved to reduce the height from 15 mm to about 6 mm. After placing about 5 g of KD in the dish for the KD-UR mice, the dish with the weighed KD was inverted and placed on the top of the food hopper. An empty water bottle was placed on top of the dish to prevent dish movement during animal feeding. The butter-like consistency adhered the KD to the inverted dish. This feeding apparatus allowed the mice easy access to the KD and prevented KD contact with bedding material. After about 24 hr, the amount of KD consumed was determined and another 5 grams of fresh KD were added to the dish. The KD was therefore given fresh every day without moving or disturbing the mice. The total amount of KD consumed per day was summed each week and divided by 7 to obtain the average weekly food intake of each mouse. For the KD-R mice, a calculated restricted amount of KD was placed directly on top of the food hopper bars for easy access. The R-fed mice licked the bars clean of the KD.

### Measurement of plasma glucose and β-hydroxybutyrate

Blood was collected approximately 1 h after seizure testing except for the pre-trial period where blood was not collected. Blood was first collected from all mice about 24 hr prior to the initiation of the 14 hr fast (Fig. 1). Mice were anesthetized with isoflurane, USP (Halocarbon, River Edge, NJ, USA) and blood was collected in heparinized tubes by puncture of the retro-orbital sinus using a borosilated capillary tube (FHC, Bowdoinham, ME, USA). The blood was centrifuged at 6,000 × g for 10 min, the plasma was collected, and aliquots were stored at -80°C until analysis. Plasma glucose concentration was measured spectrophotometrically using the Trinder Assay (Sigma-Aldrich, St. Louis, MO, USA). Plasma β-hydroxybutyrate concentration was measured using either the Stanbio β-Hydroxybutyrate LiquiColor^® ^procedure (Stanbio, Boerne, TX, USA), or a modification of the Williamson et al procedure [71].

### Statistical Analysis

Both ANOVA and a two-tailed *t *test were used to evaluate the significance of differences of body weight, seizure susceptibility, plasma glucose levels, and plasma β-hydroxybutyrate levels between unrestricted and restricted groups. Chi-square analysis was performed on the association between glucose and seizures. Pearson bivariate correlation analysis (SPSS software) was used to determine the relationship between body weight, food intake, plasma glucose levels, plasma β-hydroxybutyrate levels, and seizure susceptibility. Binary logistic regression (SPSS) was used to determine the relationship between seizure susceptibility, plasma glucose, and β-hydroxybutyrate levels on mice fed either the SD or the KD. Differences were considered significant at p < 0.01. All values are expressed as mean ± SEM. All statistical data were presented according to the recommendations of Lang et al., [72].

## Lists of abbreviations

AED, antiepileptic drug; CR, caloric restriction; KD, ketogenic diet; R, restricted; SD, standard diet; UR, unrestricted.

## Competing interests

The authors declare that they have no competing interests.

## Authors' contributions

JGM carried out all described methods and drafted the manuscript. NAC helped with the blood assays, participated in the feeding of the mice and carried out the incorporation of the data in Excel spreadsheets. MTT helped with the design of the study. RM participated in the data discussion and the statistical analysis. TNS conceived the study, participated in its design and coordination and helped prepare the manuscript. All authors read and approved the final manuscript.
